# In water, no one can hear you scream: a new species of parasitic isopod from the genus ***Zeuxokoma*** Grygier, 1993 (Isopoda: Epicaridea, Cryptoniscidae) infesting a snapping-shrimp (Caridea, Alpheidae) from Brazil

**DOI:** 10.1007/s11230-026-10300-z

**Published:** 2026-09-17

**Authors:** Amanda P. Horch, Isabela R. R. Moraes, Mariana Terossi

**Affiliations:** 1 Laboratory of Carcinology, Department of Zoology, Institute of Biosciences, Federal University of Rio Grande do Sul (UFRGS), Av. Bento Gonçalves 9500, Porto Alegre, RS 91501-970 Brazil; 2 Water Research Group, Unit for Environmental Sciences and Management, North-West University, Private Bag X6001, Potchefstroom, 2520 South Africa; 3 Programa de Pós-Graduação em Ciências Ambientais, University of Taubaté, Avenida Tiradentes, 500, Taubaté, SP 12030-180 Brazil

## Abstract

Isopods of the suborder Epicaridea Latreille, 1825 are obligatory parasites of other crustaceans, using them as intermediate and definitive hosts during their life cycles. All species of the family Cryptoniscidae Kossmann, 1880 are endoparasitic or mesoparasitic, and can be found as direct parasites of decapods or as hyperparasites of rhizocephalan barnacles infesting decapod hosts. Females usually lose all body segmentation, lacking antennae, pereopods, oostegites and pleopods, and their bodies have two portions, an anterior portion that is embedded in the host cuticle and a posterior one that is external to the host. Males, on the other hand, are neotenous, and retain the morphology of the cryptoniscus larval stage. The genus *Zeuxokoma* Grygier, 1993 comprises six valid species, all of which are direct parasites of caridean shrimps in the Atlantic and Pacific Oceans. Here we describe a new species of *Zeuxokoma* parasitizing the snapping-shrimp host *Synalpheus apioceros* Coutière from Brazil. The new species is morphologically most similar to *Z. setosa* (Nierstrasz & Brender à Brandis, 1930), but can be distinguished by the position of the shield and the insertion of the trunk. We also comment on the morphology of the last embryonic stage, provide a map detailing the distribution of all currently valid species of *Zeuxokoma*, and construct a key based on the females to assist in the identification of species. Finally, we include a table compiling the morphological characters, definitive hosts, and known distribution of the females of each species.

## Introduction

The isopods of the suborder Epicaridea Latreille, 1825 are known for being obligatory parasites of other crustaceans, which they use as intermediate and definitive hosts during their heteroxenous life cycles (Williams & Boyko, 2012). Most epicarideans go through three larval stages before attaching to their definitive hosts, with the first (epicaridium) and last (cryptoniscus) being free-living while the second stage (microniscus) is parasitic on copepods (Williams & Boyko, 2012). Epicaridea is divided into two superfamilies: Bopyroidea Rafinesque, 1815 and Cryptoniscoidea Kossmann, 1880 (Boyko et al., 2013, 2026a).

Cryptoniscoidea is the smaller of the two superfamilies, with around 100 valid species classified within twelve families (Boyko et al., 2013, 2026b). The members of this parasitic superfamily are highly modified from the free-living isopod morphology. Female cryptoniscoids are usually sac-like, and, in some families, are so greatly modified that they lack antennae, pereopods, oostegites, pleopods, and have only faintly discernible body segmentation. Males, on the other hand, are neotenous, and retain the morphology of the cryptoniscus larval stage (Williams & Boyko, 2012). Cryptoniscoids have a large variety of definitive hosts, which they can be found directly attached to as ecto-, endo-, or mesoparasites, such as amphipods, barnacles (thoracican and acrothoracican), decapods, euphausiids, isopods, mysids and ostracods (Williams & Boyko, 2012; Boyko et al., 2013). Some cryptoniscoids can be hyperparasites, living as a parasite of another parasite, and those species are usually found inside the marsupium of females from other isopod groups, including the epicaridean family Bopyridae, or attached to the externa of rhizocephalan barnacles (Williams & Boyko, 2012).

The family Cryptoniscidae Kossmann, 1880 is one of the largest within Cryptoniscoidea, composed of approximately 30 species divided into nine genera (Boyko et al., 2026c; Williams et al., 2026). All species of the family are mesoparasites, and are either found as direct parasites of decapods or as hyperparasites of rhizocephalan barnacles on decapod hosts, with the exception of *Enthylacus trivinctus* Pérez, 1920 which is endoparasitic within its rhizocephalan host (Williams & Boyko, 2012; Boyko, 2014; Boyko & Williams, 2026). Cryptoniscid females usually lose all body segmentation, lacking antennae, pereopods, oostegites and pleopods, and their bodies have two portions, an anterior portion that is embedded in the host cuticle and a posterior one that is external to the host (Williams & Boyko, 2012). Species in this family have sometimes been confused with rhizocephalan barnacles due to their morphology (Høeg & Bruce, 1988; Grygier, 1993; Boyko & Williams, 2016).

The genus *Zeuxokoma* Grygier, 1993 comprises six valid species, all of which are direct parasites of caridean shrimps in the Atlantic and Pacific Oceans (Boyko, 2014; Boyko et al., 2026c). *Zeuxokoma* has a complicated taxonomic history, having been initially described as *Zeuxo* Kossmann, 1872, a name preoccupied by the tanaidacean genus *Zeuxo* Templeton (Boyko, 2014). This would make the junior synonym *Faba* Nierstrasz & Brender à Brandis, 1930 the next available name for the genus; however, it was also already preoccupied by the mollusk genus *Faba* Fischer (Boyko, 2014). Grygier (1993) reviewed the entire history of the genus, proposed the name *Zeuxokoma*, and designated *Zeuxo alphei* Kossmann, 1872 as the type species, thereby establishing a type-species designation that had been lacking since Kossmann's (1872) original description.

Besides nomenclatural issues, there has also been some confusion regarding the morphological boundaries between the species of *Zeuxokoma* and the species of another cryptoniscid genus, *Danalia* Giard, 1887, as discussed by Boyko (2014). In the process of untangling those issues, Boyko (2014) erected a new genus, *Avada* Boyko, 2014, for some of these species. Currently, *Avada*, *Cabirnalia* Boyko & Van Der Meij, 2018, *Danalia* and *Zeuxokoma* are the only four genera in Cryptoniscidae which have a four-lobed attachment structure that is embedded within the body of the host (Boyko, 2014; Boyko & Van Der Meij, 2018; Williams et al., 2026). Unfortunately, no males or larval stages are known for any species of *Zeuxokoma*, but females of the four genera can be differentiated by their general body shapes (females of *Zeuxokoma* have relatively linear sac-shaped bodies, unlike the recurved sac-shaped bodies of *Avada*, *Cabirnalia* and *Danalia*), and their attachment lobes (strongly scleratized in *Avada* and *Zeuxokoma*, soft and flexible in *Danalia*, and short and triangular in *Cabirnalia*) (Boyko, 2014; Williams et al., 2026).

Here we describe a new species of *Zeuxokoma* parasitizing the snapping-shrimp host *Synalpheus apioceros* Coutière from Brazil, providing a morphological description of the mature female, as well as commentary on the morphology of the last embryonic stage (also called a premolt epicaridium larvae; see Williams et al., 2024 and references therein), just before the hatching of the first larval stage. We also provide a world map detailing the distribution of the other six valid species of *Zeuxokoma*, plus the new species described herein, construct a key based on the females to assist in the identification of species within the genus, and include a table compiling the morphological characters, host choice, and known distribution of each species.

## Materials and methods

**Fig. 1:**
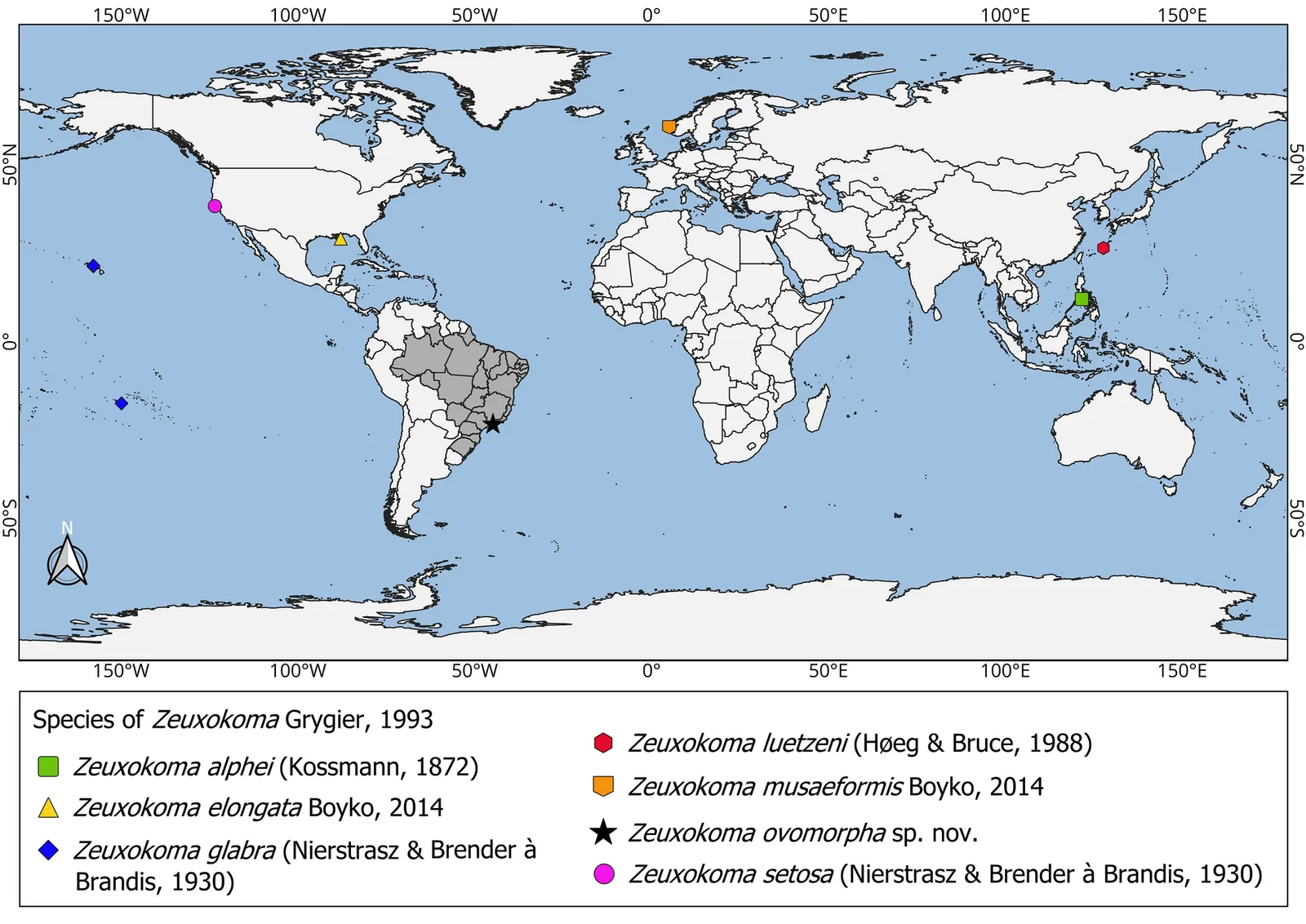
World map detailing the distribution of the seven species of the genus *Zeuxokoma* Grygier, 1993

The host and parasite specimens were collected from Ilha das Couves a continental island in Ubatuba, São Paulo, Brazil (Fig. 1), in December 2017 by SCUBA diving and passive capture, from an Artificial Refuge Substrate (ARS) at 10-meter depth. The ARS are formed by a cubic structure (25 cm (height) × 25 cm (width) × 25 cm (length), filled with artificial substrates, as a flat conduit and screens of shade nets, that remain attached on natural substrates for three months. Details of the sampling methods and the artificial substrate used are provided in Moraes et al. (2021). Both were stored together in a vial, labeled and fixed in 70% ethanol. The host shrimp and the female parasite were photographed between 10–20 times from bottom to top, using a Nikon AZ100M stereomicroscope with the software Nikon NIS-Elements AR, which were then aligned and stacked in the same software. Both host and parasite were measured through the photographs using the image processing program Fiji (Schindelin et al., 2012). The shrimp was measured for carapace length (CL), from the tip of the rostrum to the posterior margin of the carapace, and the parasite was measured for total body length (TL), maximum width, shield, and trunk length.

Line drawings of the holotype female were made using a drawing tube mounted on a Nikon SMZ800 stereomicroscope and an Olympus CX31 compound microscope. The original drawings were scanned and traced in Adobe Illustrator using a Wacom One drawing tablet. Final figures were then produced using Adobe Illustrator and Adobe Photoshop. The world map, with the location of the new species and the distribution of the previously described species of *Zeuxokoma* (Fig. 1), was made using the software QGIS (Geographic Information System; http://www.qgis.org). Occurrence data for the other six valid species of the genus were obtained from the literature (see Boyko, 2014 and references therein). Similarly, the identification key for the species of *Zeuxokoma* was based on original descriptions of the species, as well as redescriptions when necessary.

In order to obtain the embryos, a small opening was cut in the body of the female and a few embryos were carefully extracted. These specimens were prepared for scanning electron microscopy (SEM) through an ascending ethanol (EtOH) dehydration series, starting at 70% EtOH and ending at 100% EtOH. Samples were kept at each graded step between 70% and 95% for 10 minutes, then they were washed three times at 100% for 15 minutes. Subsequently, the embryos were dried using a critical point dryer (BAL-TEC CPD 030), mounted on stubs, and coated in gold (BAL-TEC SCD 050). Afterwards, specimens were viewed using a Zeiss Evo MA10 scanning electron microscope at the Center for Microscopy and Microanalysis of the Federal University of Rio Grande do Sul (CMM UFRGS).

The holotype female and the type host are deposited in the Museu de Zoologia of the Universidade de São Paulo, São Paulo, Brazil (MZUSP).

## Results

### Systematics

**Order Isopoda Latreille, 1816**

**Suborder Epicaridea Latreille, 1825**

**Superfamily Cryptoniscoidea Kossmann, 1880**

**Family Cryptoniscidae Kossmann, 1880**

**Genus *Zeuxokoma*** **Grygier, 1993**

**Type species:** *Zeuxo alphei* Kossmann, 1872 accepted as *Zeuxokoma alphei* (Kossmann, 1872) (type by subsequent designation, see Grygier, 1993).

**Diagnosis**: See Boyko (2014).

**Hosts:** Mesoparasites of caridean shrimp hosts, known to infest species of the families Alpheidae, Nematocarcinidae, Pandalidae and Thoridae (see Table 1 and references therein).

**Table 1:** Morphological characters, host choice and distribution of adult females of species in the genus *Zeuxokoma* Grygier, 1993. ATL: Atlantic Ocean; PAC: Pacific Ocean

Character	Species						
	*Z. alphei* (Kossmann, 1872)	*Z. elongata* Boyko, 2014	*Z. glabra* (Nierstrasz & Brender à Brandis, 1930)	*Z. luetzeni* (Høeg & Bruce, 1988)	*Z. musaeformis* Boyko, 2014	*Z. ovomorpha* **sp. nov.**	*Z. setosa* (Nierstrasz & Brender à Brandis, 1930)
**Body**	Very elongate	Elongate, widening distally and not recurved	Elongate and slightly recurved	Ovoid and pear-shaped, tapering towards the trunk	Elongate, slightly widening distally and slightly recurved	Compact, not recurved and slightly tapering distally	Compact and bean-shaped
**Internal segmentation**	Not described	Not described, but visible in illustrations	Visible	Faintly visible	Visible	Barely visible	Very faint
**Anteroventral shield**	Not described	Small	Shield approx. ½ length of body, restricted to anteroventral region	Not described	Small and confined to region around trunk, with 2 small ovate perforations	Small, approximately 1/5 length of body, restricted to region around trunk	Shield small and located circumanteriorly
**Trunk**	Not described	Very short	Approx. 1/2 of shield, inserted at ca. 90° angle	Approx. 1/3 the length of body	Moderately extended, inserted at ca. 90° angle	Short, approximately 1/2 length of shield, inserted at ca. 130° angle	Very short
**Point of insertion**	Region of host mouthparts	Ventrally between second and third pleomeres	Ventrolateral, close to pereopods or medioventrally on pleomeres	Ventrally in sternite of first pleomere	Pleon	Ventrally in middle of second pleomere	Not described
**Definitive Hosts**	Alpheidae: Unidentified	Nematocarcinidae: *Nematocarcinus cursor* A. Milne-Edwards	Alpheidae: Unidentified (originally cited as *Crangon* sp.), *Alpheus lobidens* De Haan and *Alpheus* sp.	Alpheidae: *Alpheus parvirostris* Dana	Pandalidae: *Pandalina profunda* Holthuis	Alpheidae: *Synalpheus apioceros* Coutière	Thoridae: *Spirontocaris holmesi* Holthuis (originally as *S. bispinosa* Holmes)
**Distribution**	PAC: Philippines	ATL: off Mississippi Delta, Gulf of Mexico, USA	PAC: Hawaii, USA; Moorea, French Polynesia	PAC: Japan	ATL: Herdla, Norway	ATL: São Paulo, Brazil	PAC: off California, USA
**References**	Kossmann, 1872; Boyko, 2014	Boyko, 2014	Nierstrasz & Brender à Brandis, 1930; Boyko, 2014	Høeg & Bruce, 1988; Boyko, 2014	Boyko, 2014	Present study	Nierstrasz & Brender à Brandis, 1930; Boyko, 2014

**Distribution:** Species found in the Atlantic and Pacific Oceans (Fig. 1; Table 1).

**Fig. 2:**
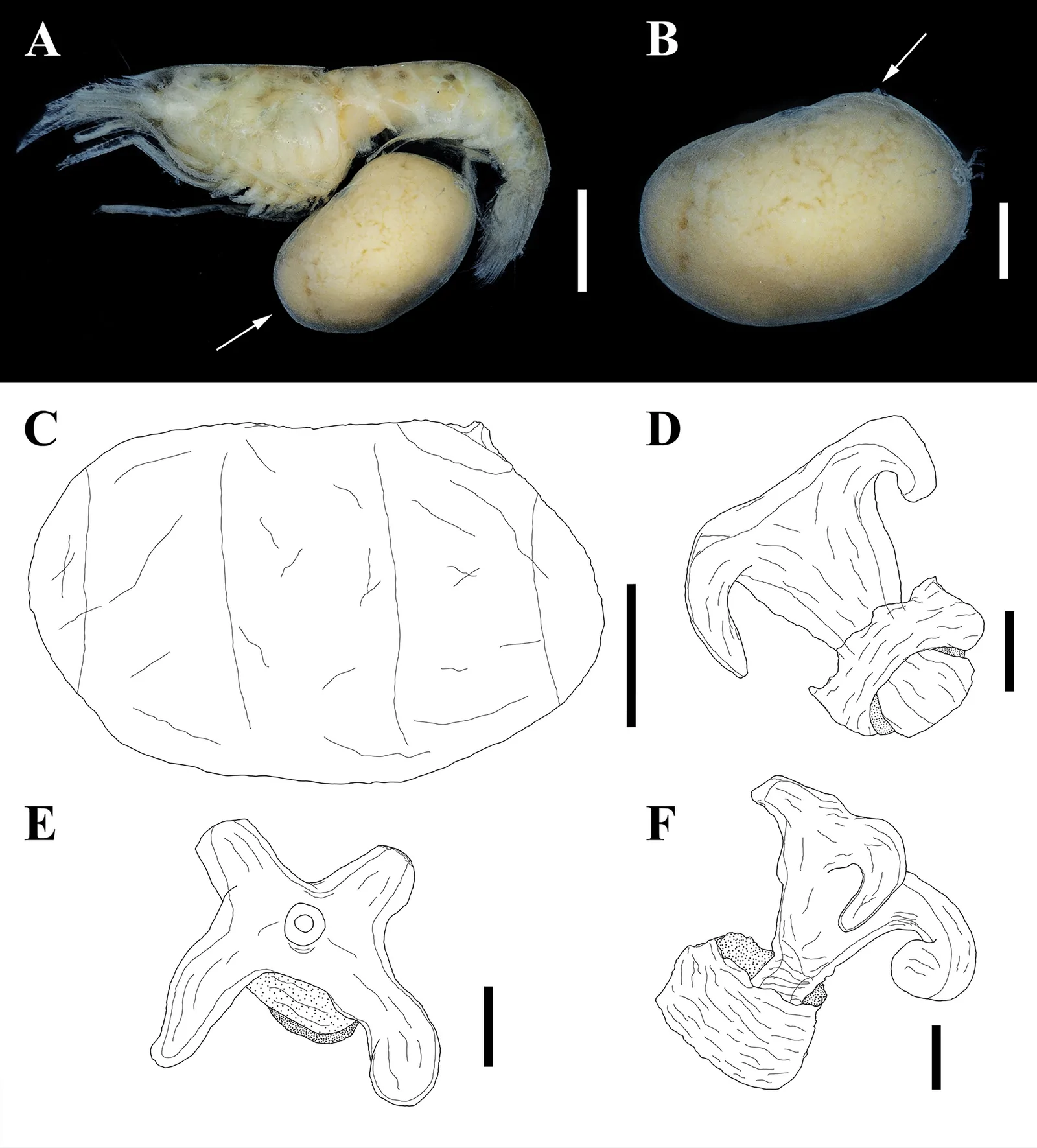
*Zeuxokoma ovomorpha* **sp. nov.**, female holotype (MZUSP 50043), and host *Synalpheus apioceros* Coutière (MZUSP 50044). A, Photograph of host shrimp in lateral view, arrow shows parasite attached to pleon; B, Photograph of holotype female habitus, lateral view, right side, arrow shows anterior region where the trunk is inserted; C, Drawing of holotype female habitus, lateral view, right side; D, Trunk, lateral view, left side; E, Trunk, dorsal view, showing the four attachment lobes and mouth, *en-face*; F, Trunk, lateral view, right side. Scales: A = 2 mm, B = 1 mm, C = 1.1 mm, D–F = 0.1 mm

***Zeuxokoma ovomorpha* sp. nov.** (Fig. 2)

**Material examined:** *Holotype*, ovigerous female (4.40 mm TL, MZUSP 50043), embedded in the pleon of a male *Synalpheus apioceros* Coutière (3.24 mm CL, MZUSP 50044), from artificial substrate, Ilha das Couves, Ubatuba, São Paulo, Brazil, 23°25'17.0"S 44°51'36.0"W, December 2017, coll. I.R.R. Moraes.

**Type host:** The caridean snapping-shrimp *Synalpheus apioceros* (Fig. 2A).

**Description:** *Mature holotype female* (Fig. 2A–F): total body length 4.40 mm, maximal body width 2.84 mm, anteroventral shield length 0.95 mm, trunk length 0.5 mm; body slightly tapering distally, longer than wide, compact, not recurved (Fig. 2A–C). Surface smooth and without lobes; no pigmentation (Fig. 2B, C). Internal segmentation only slightly visible through cuticle (Fig. 2C). Anteroventral shield small, approximately 1/5 length of body, restricted to region around trunk (Fig. 2C). Trunk short, approximately 1/2 length of anterovental shield; inserted into body at ca. 130° angle; with four recurved attachment lobes, subequal in size, two longer and thicker directed anteriorly, and two thinner and smaller directed posteriorly (Fig. 2D–F); mouth semicircular with ovate lip (Fig. 2E).

**Fig. 3:**
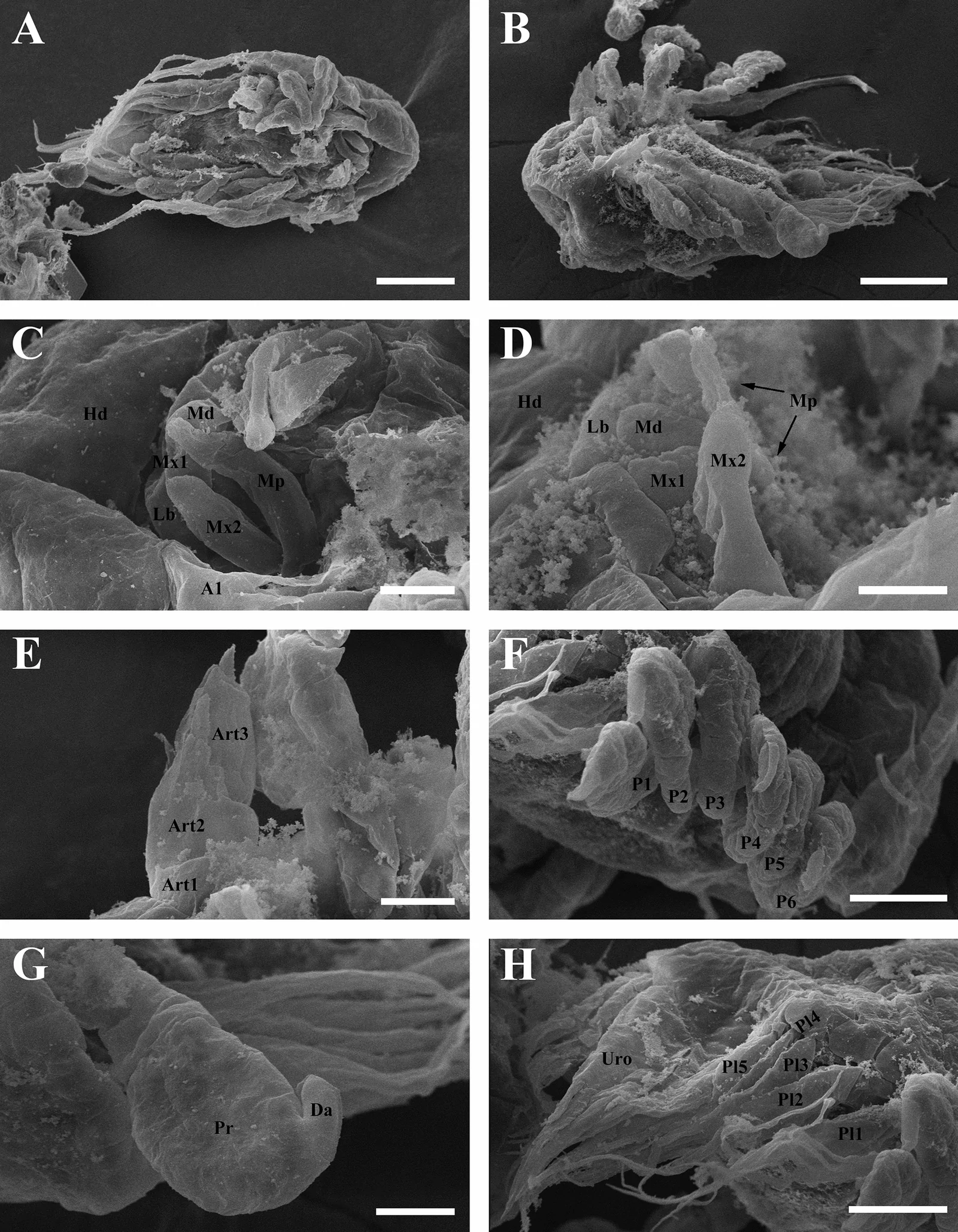
*Zeuxokoma ovomorpha* **sp. nov.**, last embryonic stage (premolt epicaridium larva) from female holotype (MZUSP 50043) in scanning electron microscopy. A, Ventral view; B, Lateral view; C, Ventral view of head, showing antennule and mouthparts; D, Lateral view of mouthparts; E, Right antennule; F, Pereopods; G, Detail of left pereopod 6; H, Pleopods and uropod. Abbreviations: Hd, head; A1, antennule; Lb, labrum; Md, mandible; Mx1, maxillula; Mx2, maxilla; Mp, maxilliped; Art1–3, articles 1–3; P1–P6, pereopods 1–6; Pr, propodus; Da, dactylus; Pl1–Pl5, pleopods 1–5; Uro, uropod. Scales: A, B = 40 μm, C, G = 9 μm, D = 6 μm, E = 8 μm, F, H = 20 μm

*Description of last embryonic stage (premolt epicaridium larva):* Body egg-shaped (Fig. 3A, B), approximately 185 μm long from anterior margin of head to end of uropods. Final articulation of most appendages not clearly distinguished.

Anterior margin of head rounded (Fig. 3A). Antennules (Fig. 3E) short, composed of three articles each; second article with single stout setae distolaterally, terminal article with single stout seta distally. Antennae (Fig. 3A, B) long, nearly full length of body, with two simple terminal setae (Fig. 3B). Mouthparts (Fig. 3C, D): mandibles rounded; maxillula (= maxilla 1) similar in size and rounded; maxilla (= maxilla 2) slightly longer than maxillula, conical; maxilliped longer than maxilla, conical and narrowing towards the distal end.

Six pairs of gnathopodal pereopods (Fig. 3F), subequal in size and shape. Only articulation of propodus and dactylus can be distinguished; other articles obscured (Fig. 3G). Pleon with five pairs of birramous pleopods (Fig. 3H), articulation indistinguishable. Uropods birramous (Fig. 3H), rectangular peduncle with well-developed short seta distolaterally; slender endopod and exopod, each with one simple, short, stout seta at the distal end.

**Distribution:** Known only from the type locality in Ilha das Couves (23°25'17.0" S 44°51'36.0" W), Ubatuba, São Paulo, Brazil (Fig. 1).

**Etymology:** Named *ovomorpha* due to the similar egg-like body shape of the mature female and the first life stage of the xenomorph alien characters in the 1979 *Alien* film and media franchise, known as “ovomorph”. As well as the resemblance of the parasitic female attachment lobes and the four petals of the ovomorphs, which open to release the second life stage of the alien, the “facehugger”. Although it is a visual resemblance only, since the lobes of the cryptoniscid females function as attachment structures to the host and do not release larvae.

**Remarks:**

The holotype female was attached to the host shrimp ventrally in the second pleomere, approximately in the middle, with the four lobes of the attachment process inserted into the host cuticle (Fig. 2A). The new species is morphologically most similar to *Z. setosa* (Nierstrasz & Brender à Brandis, 1930) from the Pacific Ocean off California, USA, with very limited segmentation visible, small anteroventral shield and short trunk (Boyko, 2014). But the female of *Z. ovomorpha* **sp. nov.** can be distinguished from *Z. setosa* by the position of the shield (anteroventral in the new species vs. circumanteriorly in *Z. setosa*) and the insertion of the trunk (ca. 130° in the new species vs. ca. 90° in *Z. setosa*). The species *Z. elongata* Boyko, 2014 also has a short trunk (see Boyko, 2014) similar to *Z. ovomorpha* **sp. nov.**; however, the overall body shape of *Z. elongata* is much more elongated than that of *Z. ovomorpha* **sp. nov.**, as well as more recurved, and they have different host attachment locations (between the second and the third pleomeres in *Z. elongata*). For more in-depth comparisons between all *Zeuxokoma* species see Table 1. No cryptoniscus larvae or neotenous males were found associated with the holotype female or the host; so, unfortunately, those life stages could not be described.

**Comments on last embryonic stage (premolt epicaridium larva):** The holotype female is ovigerous, carrying embryos in the last embryonic stage (Fig. 3A–H) before becoming the free-living epicaridium larvae and being released into the water column. This last embryonic stage is referred to as a premolt epicaridium larva by Williams et al. (2024), where they describe this life stage for the bopyroid species *Pleurocryptella poseidon* Williams & Boyko in Williams, Boyko & Stewart, 2024, and compare the morphology of epicaridium larvae within Epicaridea, with a focus on the differentiation of the mouthparts.

Goudeau (1977) recorded and named the embryonic stages of the hemioniscid *Hemioniscus balani* Buchholz, 1866, parasitizing the thoracic barnacle *Austrominius modestus* (Darwin) in France, and observed that the embryos undergo two embryonic molts during their development inside the female marsupium. According to the stages proposed by Goudeau (1977), the last stage, called ‘stage J’, is composed of the embryonic membranes 3 and 4, plus a membrane 5, which is the formed epicaridium larva. Having already undergone one molt and the release of membrane 1, the embryo undergoes another molt where it sheds membranes 3 and 4, hatching into an epicaridium larva and leaving the marsupium (Goudeau, 1977). Bocquet-Védrine (1987) recorded a similar embryonic development in the crioniscid *Crinoniscus equitans* Pérez, 1900, a parasite of the thoracic barnacle *Perforatus perforatus* (Bruguière) from Arcachon Basin, France. She observed, however, that the *C. equitans* embryos in the last stage were only surrounded by two membranes: membrane 4, in which the morphology of the embryo is already differentiated, and consists of fully formed appendages and sensory structures; and membrane 5, which has formed the final epicaridium morphology (Bocquet-Védrine, 1987). This differs from Goudeau’s (1977) observations in *H. balani*, but matches what we observed here in the embryos of *Z. ovomorpha* **sp. nov.** (Fig. 3A–H), which we consider to be in this last embryonic stage or be at a premolt epicaridium larva stage (*sensu* Williams et al., 2024), with formed appendages and sensory structures similar to those observed by Bocquet-Védrine (1987) for *C. equitans*.

By comparing the morphology of the last embryonic stage with the fully hatched epicaridium larva of *C. equitans*, Bocquet-Védrine (1987) concluded that the most relevant morphological differences between them could be found in the antennules and the uropods. The antennules of the *Z. ovomorpha* **sp. nov.** embryos (Fig. 3E) are similar to those of *C. equitans* (see fig. 1A of Bocquet-Védrine, 1987), in that they are composed of three articles, and the third article has setae distally. They differ in the number of setae observed in article 3: *Z. ovomorpha* **sp. nov.** has a single stout seta at the distal end (Fig. 3E), and *C. equitans* has two smaller ones (Bocquet-Védrine, 1987). The uropods (Fig. 3H) also have a similar overall morphology to *C. equitans* (see fig. 5A of Bocquet-Védrine, 1987), however, the uropods of the *Z. ovomorpha* **sp. nov.** embryos appear to have a longer and more rectangular peduncle than *C. equitans*, as well as longer setae (Fig. 3H).

## Discussion

*Zeuxokoma ovomorpha* **sp. nov.** is the seventh species to be described in the cryptoniscid genus *Zeuxokoma* (Boyko, 2014), as well as the first species of the genus recorded in the South Atlantic Ocean (Fig. 1). The only two other species of *Zeuxokoma* currently known from the Atlantic Ocean are (see Fig. 1): *Z. elongata* Boyko, 2014, parasitizing the nematocarcinid shrimp *Nematocarcinus cursor* A. Milne Edwards, off the Mississippi Delta, USA, within the Gulf of Mexico in the Western Atlantic, and *Z. musaeformis* Boyko, 2014, parasitizing the pandalid *Pandalina profunda* Holthuis, from Herdla, Norway, in the Eastern Atlantic (Boyko, 2014). More regionally, *Z. ovomorpha* **sp. nov.** is the second species of the family Cryptoniscidae to be described in Brazil, following *Cryptoniscus planarioides* Müller, 1864, a hyperparasite of the rhizocephalan *Peltogster purpureus* (Müller) from a hermit crab host, which was described more than a century earlier, and raises the number of species of the entire superfamily Cryptoniscoidea in the country to five (Ribeiro & Horch, 2023). When considering the definitive host, this is the first record of a species of *Zeuxokoma* parasitizing a shrimp of the genus *Synalpheus* Spence Bate, although three other species of the genus are known to occur as parasites of other closely related alpheid genera, namely *Alpheus* Fabricius (see Table 1 and references therein).

Cryptoniscoids appear to be rare in general, and get less attention in the literature when compared to the bopyroids, especially the ectoparasitic bopyrids (Boyko & Williams, 2016). This is likely due to their sac-like morphology, which is nearly devoid of typical isopod characters, and the cryptic nature of many species as hyperparasites or endoparasites (Williams & Boyko, 2012). Beyond the now seven species described in *Zeuxokoma*, Boyko (2014) also cited two undescribed species that could potentially also belong to the genus: *Zeuxokoma* sp. van Kampen & Boschma, 1925 parasitizing *Alpheus malleodigitus* (Spence Bate) from the Philippines, although it should be noted that, as suggested by Boyko (2014), the semi-spherical body shape of this species makes its proposed placement in *Zeuxokoma* questionable, and *Zeuxokoma* sp. Boyko, 2014, parasitizing *Cinetorhynchus hendersoni* (Kemp) from Japan. These, alongside recent studies describing new species from other genera of Cryptoniscidae (Boyko & Van Der Meij, 2018; Williams et al., 2026), show there are likely more species of both *Zeuxokoma* and Cryptoniscidae in general to be discovered and described worldwide.

There is also a lack of larval and male descriptions in Cryptoniscidae, with many genera only being known from the female form, as is the case with *Zeuxokoma* (Boyko, 2014). Here, the male specimen could have been lost during the sorting of the artificial substrate or posterior specimen manipulation prior to the samples' arrival at the laboratory. Despite that, we were able to observe the last embryonic stage of *Z. ovomorpha* **sp. nov.**, a first for the genus. It is clear that more research is needed to better understand the real diversity of cryptoniscid species, as well as their phylogenetic relationships with each other and with other cryptoniscoids.

## Key to species of *Zeuxokoma* based on adult females

1. Body shape elongated............................................................................................................ 2

- Body shape compact or ovoid................................................................................................. 5

2. Attached to the host pleon, either ventrally or dorsally.....................................................﻿.... 3

- Attached to the region of the host mouthparts .................................................*Z. alphei* (Kossmann, 1872)

3. Shield size approximately ½ length of body........................ *Z. glabra* (Nierstrasz & Brender à Brandis, 1930)

- Shield size small, less than ½ length of body.......................................................................... 4

4. Trunk very short.................................................................................................*Z. elongata* Boyko, 2014

- Trunk moderately extended, inserted at ca. 90° angle................ *Z. musaeformis* Boyko, 2014

5. Trunk size approximately 1/3 the length of the body.....................................*Z. luetzeni* (Høeg & Bruce, 1988)

- Trunk size small or approximately 1/2 the length of the body or less................................ 6

6. Body bean-shaped, with small shield located circumanteriorly.................................*Z. setosa* (Nierstrasz & Brender à Brandis, 1930)

- Body not recurved and slightly tapering distally, with small shield restricted to region around trunk................................ *Z. ovomorpha* **sp. nov.**

## Data Availability

Type material and all specimens examined are deposited in the Museu de Zoologia of the Universidade de São Paulo, São Paulo, Brazil (MZUSP), under the voucher numbers MZUSP 50043 and 50044, and are available for study.
